# Availability of healthy and unhealthy foods in modern retail outlets located in selected districts of Greater Accra Region, Ghana

**DOI:** 10.3389/fpubh.2022.922447

**Published:** 2022-11-09

**Authors:** Akosua Pokua Adjei, Gideon Senyo Amevinya, Wilhemina Quarpong, Akua Tandoh, Richmond Aryeetey, Michelle Holdsworth, Charles Agyemang, Francis Zotor, Matilda E. Laar, Kobby Mensah, Phyllis Addo, Dennis Laryea, Gershim Asiki, Daniel Sellen, Stefanie Vandevijvere, Amos Laar

**Affiliations:** ^1^Department of Population, Family and Reproductive Health, School of Public Health, University of Ghana, Accra, Ghana; ^2^Nutrition and Health Sciences, Laney Graduate School, Emory University, Atlanta, GA, United States; ^3^UMR MoISA (Montpellier Interdisciplinary Centre on Sustainable Agri-Food Systems), (Univ Montpellier, CIRAD, CIHEAM-IAMM, INRAE, Institut Agro, IRD), Montpellier, France; ^4^Department of Public and Occupational Health, Amsterdam UMC, University of Amsterdam, Amsterdam, Netherlands; ^5^Department of Family and Community Health, University of Health and Allied Sciences, Ho, Ghana; ^6^Department Family and Consumer Sciences, School of Agriculture, University of Ghana, Accra, Ghana; ^7^Department of Marketing and Entrepreneurship, University of Ghana Business School, University of Ghana, Accra, Ghana; ^8^Non-Communicable Disease Programme, Ghana Health Service, Accra, Ghana; ^9^African Population and Health Research Center, Nairobi, Kenya; ^10^Department of Nutritional Sciences, University of Toronto, Toronto, ON, Canada; ^11^Sciensano, Service of Lifestyle and Chronic Diseases, Brussels, Belgium

**Keywords:** ultra-processed food, modern retail outlets, supermarket, non-communicable diseases, Ghana

## Abstract

**Background:**

Intake of unhealthy foods is linked to the onset of obesity and diet-related non-communicable diseases (NCDs). Availability of unhealthy (nutritionally poor) foods can influence preference, purchasing and consumption of such foods. This study determined the healthiness of foods sold at modern retail outlets- supermarkets and mini-marts in the Greater Accra Region of Ghana.

**Methods:**

All modern retail outlets located in six districts of Greater Accra were eligible. Those < 200 m^2^ of floor area and with permanent structures were categorized as mini-marts; and those ≥200 m^2^ as supermarkets. Shelf length of all available foods were measured. Healthiness of food was determined using two criteria - the NOVA classification and energy density of foods. Thus, ultra-processed foods or food items with >225 kcal/100 g were classified as unhealthy. The ratio of the area occupied by unhealthy to healthy foods was used to determine the healthiness of modern retail outlets.

**Results:**

Of 67 retail outlets assessed, 86.6% were mini-marts. 85.0% of the total SHELF area was occupied by foods categorized as unhealthy (ranging from 9,262 m^2^ in Ashiaman Municipality to 41,892 m^2^ in Accra Metropolis). Refined grains/grain products were the most available, occupying 30.0% of the total food shelf space, followed by sugar-sweetened beverages (20.1% of total shelf space). The least available food group–unprocessed staples, was found in only one high income district, and occupied 0.1% of the total food shelf space. Retail outlets in two districts did not sell fresh fruits or fresh/unsalted canned vegetables. About two-thirds of food products available (*n* = 3,952) were ultra-processed. Overall, the ratio of ultra-processed-to-unprocessed foods ranged from 3 to 7 with an average (SD) of 5(2). Thus, for every healthy food, there were five ultra-processed ones in the studied retail outlets.

**Conclusion:**

This study reveals widespread availability of ultra-processed foods in modern retail outlets within the selected districts. Toward a healthier food retail environment, public health and food regulators, in partnership with other stakeholders need to institute measures that improve availability of healthy foods within supermarkets and mini-marts.

## Introduction

Obesity is a major public health issue that contributes to the development of other diet-related non-communicable diseases (NCDs) such as diabetes, hypertension, cardiovascular disease (CVD) and some cancers (1). Several policies and programmes have been recommended to limit the intake of unhealthy ultra-processed, energy-dense and nutrient-poor foods rich in calories, salt, sugar, and fat but poor in beneficial nutrients such as fiber, protein, and micronutrients (2, 3). This policy response to improve food environments are informed by evidence that suggests that availability of these foods in the individual's environment and individual factors such as attitudes, taste, income and food affordability influence food consumption, including within Africa (4, 5). This implies that limiting the availability of unhealthy foods, while increasing the availability of healthier foods can motivate healthier food acquisition or consumption, and may thus contribute to preventing obesity. To that end, many calls exist for creating and promoting healthier food environments while eliminating so called “obesogenic environments” that encourage unhealthy food consumption (5, 6).

Urbanization, poverty, globalization, industrialization, climate change and the emergence of a concentrated corporate food regime (1–3) are converging and mutually reinforcing global transitions such as the dietary transition (4). In Ghana, such transformations of global food systems understood as the web of processes, actors and infrastructure that govern food production, processing, distribution, and sale have contributed to a changing quality of diets and the rise in obesity and nutrition-related NCDs (5). Ghana is at an advanced stage of the nutrition transition, experiencing rapid urbanization, and increasing overweight/obesity and related NCDs (5, 6). This entails increasing consumption of ultra-processed, energy-dense and micronutrient-poor foods, compounded by sedentary lifestyles with reduced physical activity. The transformation of systems of food production toward industrial mass-production of ultra-processed food has been mirrored by other structural changes including the transformation of food retail (2).

Ghana has recently been ranked as highly attractive for retail investment in Africa and globally based on the Global Retail Development Index, which studies the global retailing landscape based on 25 criteria (including country risk, market attractiveness, market saturation, and national retail sales) (7). Ghana has also been described as the new “bright spot” of Africa, driven by increased foreign and public investment as well as urbanization of the population. As a result, modern retail in Ghana is gradually emerging, especially in the capital, Accra (7), therefore impacting on food environments. These changes have included more direct contractual connections between large-scale producers and suppliers, with centralized and consolidated procurement and distribution systems. These “upstream” trends are mirrored by the “downstream” diffusion and penetration of “supermarkets for the poor” into areas previously dominated by traditional and informal markets (8). In Ghana, not only has the number of modern retail outlets increased, but also the penetration of ultra-processed foods in traditional food retail outlets—including informal ones (9). Monteiro et al. (3), defined ultra-processed foods as industrial products consisting of various processed ingredients, including sugar, starches, emulsifiers or colors, salt and other additives that enhance the shelf life or modify the food's taste, texture and color. Typical examples consumed in Ghana are sweets and biscuits, fried sausage and instant noodles (10).

The NOVA framework categorizes food into four groups (ultra-processed foods and three others) based on their nature, purpose, and degree of processing (3). Ultra-processed food consumption reduces the likelihood of consuming fiber, protein and many micronutrient-rich foods that promote wellbeing, while increasing free sugar, sodium and unhealthy fats intake (3). Several studies have demonstrated a correlation between consumption of ultra-processed foods and increased risk for obesity and other NCDs (11–13). These studies and others indicate that not only are they available in retail outlets, they are also widely marketed, including in Ghana (14–16). Such wide availability and concerted marketing of ultra-processed foods a role in the purchasing and consumption of these foods (17).

Shelf space has been shown to have a substantial influence on the sales of a variety of food items (both healthy and unhealthy), indicating that the accessibility and prominence of potentially unhealthy foods in the supermarket, such as sugar sweetened beverages and salty snacks can also have an impact on the healthiness of customers' diets (18). Likewise, a classic marketing study showed that with a doubling of shelf space, sales of fruits and vegetables rose by about 40% (19). Elsewhere, studies that aimed to assess the healthiness of supermarket food environments have measured the shelf length of foods available for sale in food retail outlets and categorized them as healthy or unhealthy [e.g., (20, 21)]. These studies showed that simple measurements of shelf space can be used by researchers to characterize the healthfulness of the food environment and by policymakers to establish criteria for favorable policy treatment of stores (20, 21). Vandevijvere et al. (21) for instance reported that for every 1 m of shelf length for unhealthy foods, there was 42 cm of shelf length for healthy foods on average, with large variations between stores. The shelf length ratio was significantly lower in the most compared to the least/medium deprived socioeconomic areas.

In Ghana, while research on food retail environments is increasing in both scope and level of innovation (6, 15, 16, 22, 23), none has measured the amount of shelf space used for healthy and unhealthy foods in-store. Shelf space measurement is a useful measure of the in-store availability of healthy vs. less foods. The measure is a good proxy for product availability in food retail outlets. The current study determined the availability of food types sold at modern retail outlets according to their level and purpose of food processing and energy density through measuring shelf length located in the Greater Accra Region of Ghana.

## Methods

### Study design and site

**Figure 1:**
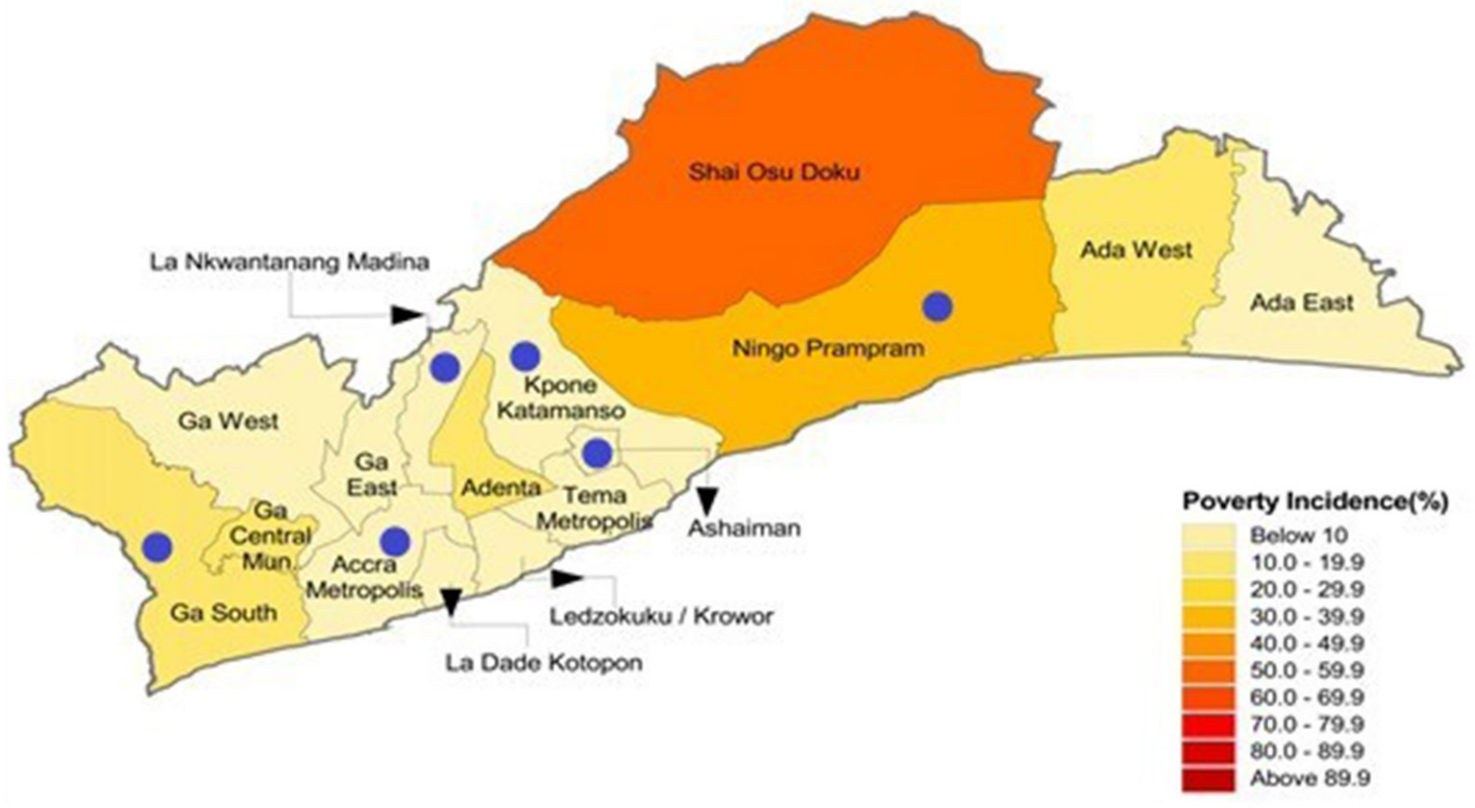
A map of the Greater Accra Region of Ghana highlighting the six selected districts and their poverty incidence.

This study is part of the Measurement, Evaluation, Accountability, and Leadership Support for NCDs Prevention Study (MEALS4NCDs) Project, which was implemented in the Greater Accra Region (GAR) of Ghana in 2019-2022 (24). MEALS4NCDs's overarching aim was to measure and support public sector actions that create healthy food marketing, retail, and provisioning environments for Ghanaian children, using adapted methods from the International Network for Food and Obesity/NCDs Research Monitoring and Action Support (INFORMAS) (25). The MEALS4NCDs project adopted a cross-sectional study design that applied both quantitative and qualitative methods. The study deployed a multistage sampling approach to select six administrative districts in the Greater Accra Region of Ghana (Figure 1). The first stage of the sampling process entailed purposively selecting the Greater Accra Region, which hosts the national capital, was/and remains the most urbanized and most marketed to, region of Ghana. The region was sub-divided into 16 administrative districts categorized as Metropolitan, Municipal, and Districts Assemblies (MMDAs). A representative sample of six districts (one metropolitan, three municipals and two districts) were selected using both probabilistic and non-probabilistic sampling approaches. Details about the sampling is reported elsewhere (24).

All modern retail outlets located in these six districts were eligible to participate in the study.

### Modern food retail outlet identification

An eligible “retail outlet” was defined as a self-service modern retail outlet (with aisles accessible to customers) that had at least one staffed checkout aisle or cash register. In this study, “modern food retail outlets” encompass supermarkets and mini-marts. All eligible ‘retail outlets with < 200 m^2^ of floor area were categorized as a “mini-mart” as opposed to supermarkets that were much larger in size. Of note, the 200 m^2^ criterion was not decided a priori. Its relevance came to the fore when the need arose to discriminate between usual supermarkets and “smaller marts/hypermarkets that were included in the assessment.” The derivation of the criterion was informed by the study team's collective knowledge and lived experiences. The criterion was deemed sensitive enough to discriminate between these two kinds of outlets. While local district offices were able to provide list of modern retail outlets found within their jurisdictions, field data collectors were trained to identify and map those that may not have been captured in the district database.

### Summary of field procedures

Data were collected from July to August, 2020. Permission was obtained from retail outlet managers and owners to conduct in-store measurements. An in-store measurement tool adapted from INFORMAS was used to determine the total shelf length/breadth of foods available in the supermarkets—consistent with previous studies (20, 26). The shelf length and breadth for all food groups were measured: sugar-sweetened beverages, refined grains and refined grain products, whole grain cereal, legumes, fresh meat, processed meat/fish, milk product, eggs, fresh fruits and fresh fruit juice, fresh vegetables, fats and oil products, water, alcoholic beverages, unsweetened coffee, sweet foods and toffees, salted snack and vegetables, unprocessed staples, processed staples and condiments. When a food item type (e.g., sugar-sweetened beverage) was found in several places in the retail outlet, each location was measured separately and recorded in the in-store measurement tool. The total shelf area was added, once all measurements had been conducted. The total floor space was also determined by measuring the retail outlet's floor length and breadth. Digital photographs of all food products in-store were taken to facilitate their classification.

Each district had a team of two trained research assistants who conducted the in-store assessment (shelf space measurement with a tape measure and taking photographs). The research assistants went through several workshops to ensure clarity and consistency of comprehension. The data collection tool was pretested and validated before being used finally to collect data. The pretesting was done in La Nkwatanang-Madina Municipal Assembly. Based on the results obtained from the pretesting, necessary updates were done to the data collection tool.

### Healthiness of food products

The foods were categorized according to the extent and nature of processing using the NOVA classification system (3). The NOVA food classification model categorizes foods into four groups—unprocessed/minimally processed foods, processed culinary ingredients, processed foods, and ultra-processed foods. In this study, unprocessed or minimally processed food were deemed healthy, whilst ultra-processed foods were not. Given our interest in calorie density of foods, food products were secondly categorized as healthy or unhealthy foods depending on calorie density. The NOVA classification does not specifically classify foods using calorie density. Foods that were energy-dense (>225 kcal/100 g) according to the (27) classification, were nutrient-poor, or were high in free/added sugar, sodium, and saturated fat were classified as unhealthy; healthy foods on the other hand were those that are nutrient dense. Details of the process of categorizing are given in our previously published paper (10). Other food categories including condiments/culinary ingredients were classified as miscellaneous. For every retail outlet we computed the ratio of all unhealthy to all healthy products, as well as ultra-processed food to minimally processed/unprocessed foods.

### Healthiness of retail outlets

The ratio of total area of shelf space occupied by unhealthy vs. healthy food types available in retail outlets was calculated. In this study, a modern food retail outlet with a ratio less than one (< 1) was considered to be healthier; a ratio greater than or equal to one (≥1) was considered less healthy. Similarly, an outlet was scored based on the ratio of NOVA Class four (ultra-processed food) to NOVA Class one (unprocessed/minimally processed). Again, a ratio < 1 was considered as a healthier supermarket (< 1). A ratio ≥1 was considered less healthy.

### Data analysis

Descriptive analyses were performed using Microsoft Excel Worksheet 2010 and IBM SPSS Version 21 to generate total areas occupied by the various food groups and the ratio of healthy to unhealthy foods. Descriptive statistics such as means, standard deviations and percentages were estimated for the features of the retail outlets and the relative availability of unhealthy foods.

## Results

Within the six districts selected, 113 eligible modern food retail outlets were identified. Assessments were conducted in 67/113 (59%) of the food outlets in operation. Assessments were not conducted in the remaining retail outlets primarily because their managers did not grant permission for measurements or for pictures to be taken. Also, a few others were not operating and could not be accessed. The attributes of the retail outlets are shown in Table 1. We adopted Ghana Statistical Service's categorization of the districts by poverty incidence (also referred to as poverty headcount and defined in Table 1). La Nkwatanang Madina (LANMA) had the highest number of retail outlets assessed (22) and also the highest mean outlet floor size (207 m^2^). The sizes of the retail outlets differed by district. 86.6% of the retail outlets assessed were mini-marts (floor area < 200 m^2^). About 80% of the retail outlets were found in districts with low poverty incidence (≤ 10% poverty incidence). The average floor size of the supermarkets found in LANMA, one of the districts in the Greater Accra Region with low poverty headcount (2.8%) was 207 m^2^, followed by AMA (104 m^2^) whilst Ningo Prampram, a district with high poverty headcount - more than four times the regional average (poverty headcount of 31.2%), had an average outlet floor size of 63 m^2^. Ga South Municipal, a district with the highest number of poor persons (61,347) in the region had the least average supermarket floor size (46 m^2^).

**Table 1:** Attributes of identified retail outlets located within the study districts.

**District**	**Number of retail outlets identified (N)**	**Number of retail outlets assessed (%)**	**Range of retail outlets area (m^2^)**	**Mean retail outlet area (m^2^)**	**Mini marts (%)**	**Supermarkets (%)**	Poverty headcount*(%)	**Poverty profile**
Accra Metro	24	58.3	13–334	104	78.6	21.4	2.6	Low
Ashaiman	10	60.0	28–109	63	100.0	0.0	4.4	Low
Ga South	11	81.8	16–91	46	100.0	0.0	15.2	High
La Nkwantanang	32	68.8	26–864	207	72.7)	27.3	2.8	Low
Kpone Katamanso	22	27.3	24–181	85	100.0	0.0	3.5	Low
Ningo Prampram	14	71.4)	26-90	63	100.0	0.0	31.2	High

*Poverty incidence or headcount is defined as the ratio of the number of people in a district whose income falls below the poverty line. Low poverty incidence ≤ 10% poverty incidence.

### Food items available in the modern retail outlets

**Figure 2:**
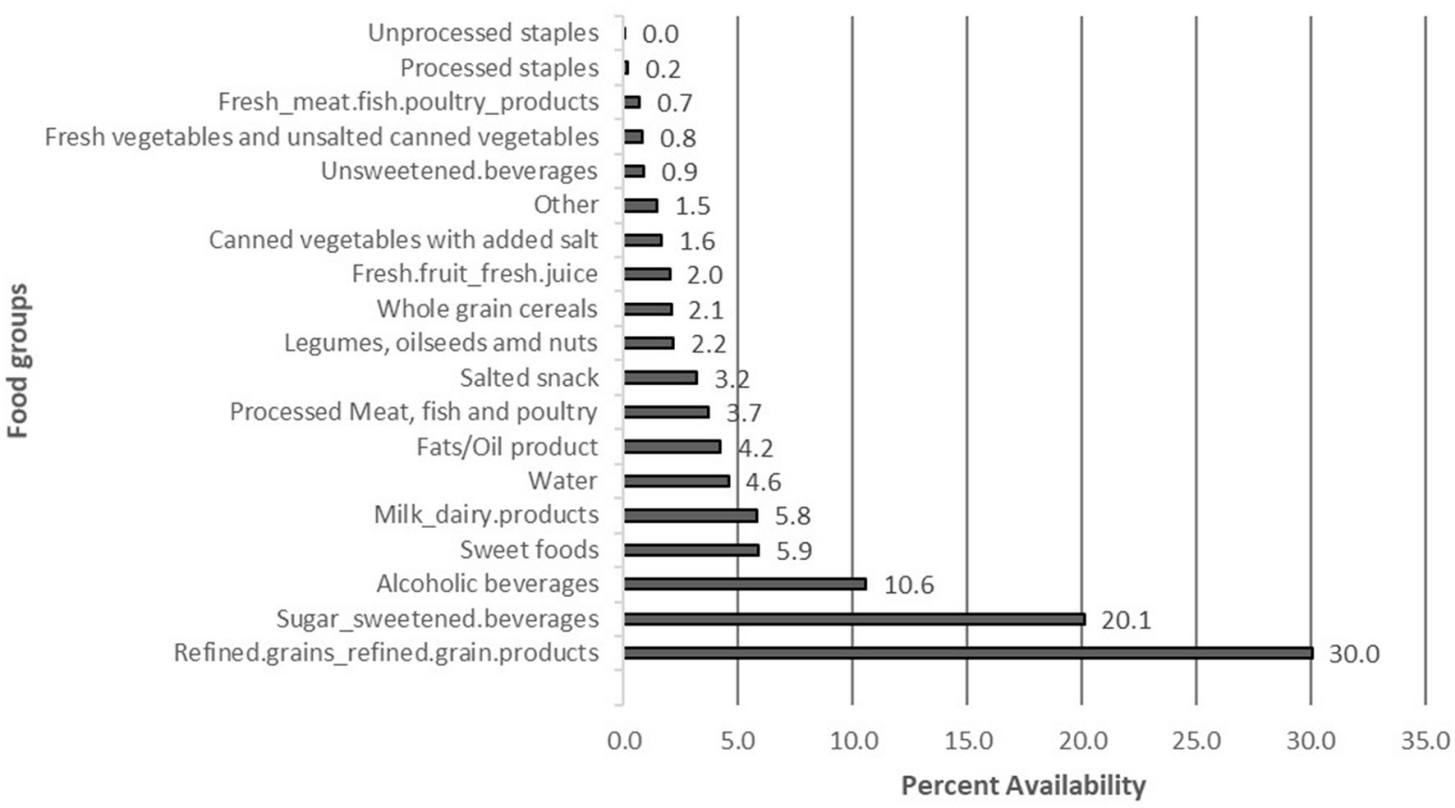
Availability (measured by proportion of total shelf area [%]) of different types of food items in modern retail outlets within the study districts.

Refined grain/grain products such as noodles, cornflakes, biscuits, cake and cookies were the most available food groups in the retail outlets within the selected districts, except Ga South, where alcoholic beverages occupied the majority of the shelf area dedicated to foods in retail outlets (40.0%) (Figure 2). This was followed by sugar-sweetened beverages in all the districts except Ga South, where refined grains and refined grain products were the second most available food group (23%). Fresh vegetables and unsalted canned vegetables were not found in any of the retail outlets assessed in Ashaiman. No retail outlet assessed in Ga South sold fresh fruit. Additional data (Appendix 1) shows the availability [measured by shelf area (m^2^)] of the different food items in the retail outlets assessed in the six districts.

### Availability of healthy vs. unhealthy foods

**Figure 3:**
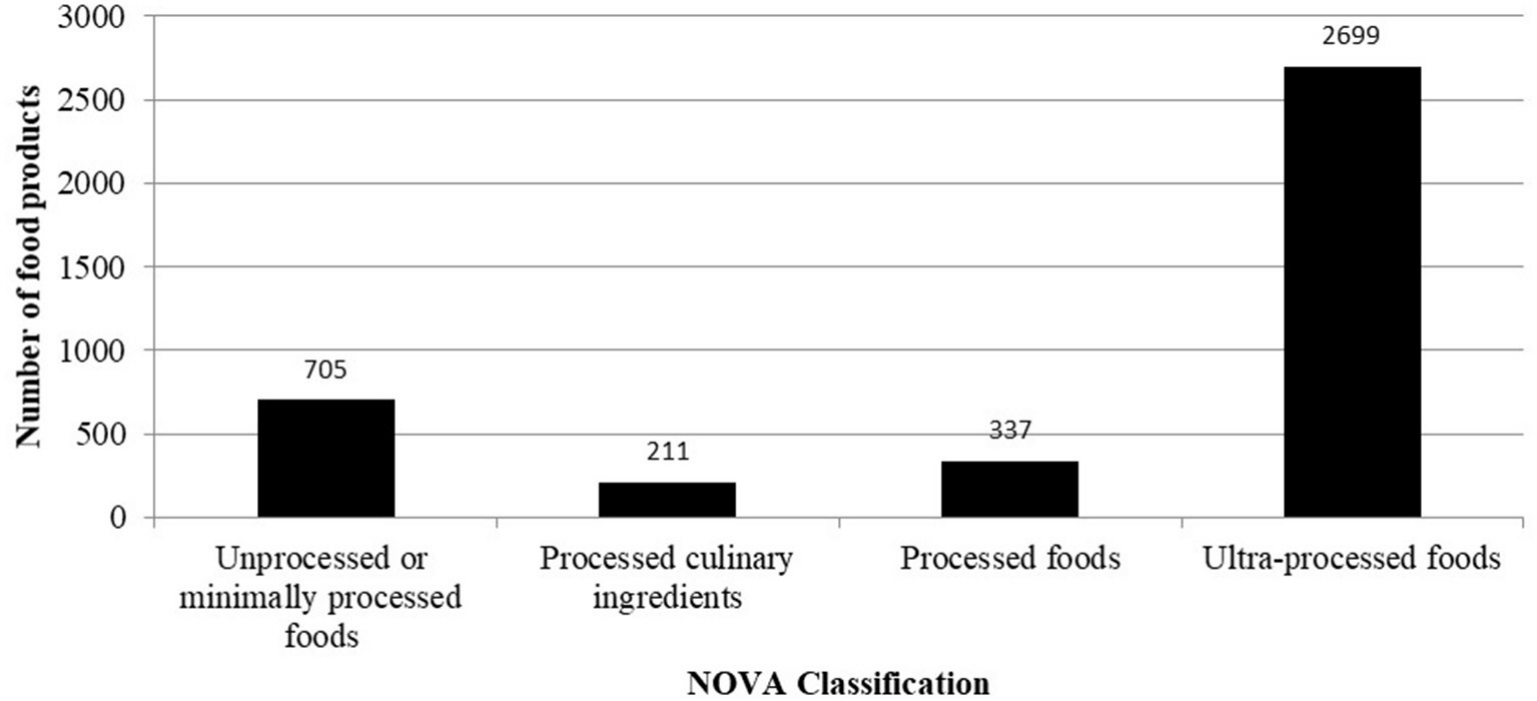
NOVA classification of unique food products in modern food outlets in all six study districts.

Over eighty percent (83.1%) of the shelf space occupied by healthy foods were found in retail outlets located in low poverty incidence districts (Table 2). Overall, 78.7% of the area occupied by food products in both the mini-marts and supermarkets was allocated to “unhealthy” foods; about 20% of these were sugar-sweetened beverages (Appendix 1). Using the NOVA classification system, 68% (2669) food products identified in the food outlets were categorized as ultra-processed, 9% (337) were processed foods, 5% (211) were processed culinary foods and 18% (705) were unprocessed or minimally processed foods (Figure 3).

**Table 2:** Shelf area occupied by healthy and unhealthy foods in retail outlets by districts, outlet size, and district poverty level.

**Characteristic**	**Healthy foods (m^2^)** **Mean (SD)**	**Unhealthy foods (m^2^)** **Total mean (SD)**	**Miscellaneous/culinary ingredients (m^2^)** **Total mean (SD)**
**District**			
Accra Metro	973 (707)	2992 (2472)	143 (144)
Ashiaman	250 (110)	1547 (397)	12 (19)
Ga South	230 (82)	1667 (1678)	13 (12)
La Nkwantanang	872 (1602)	4557 (6886)	310 (582)
Kpone katamanso	1300 (2887)	3027 (6078)	2 (5)
Ningo Prampram	585 (1097)	2404 (1718)	10 (26)
**Outlet type**			
Mini-mart (m^2^)	618 (1024)	2159 (2295)	52 (89)
Supermarket (m^2^)	1818 (2445)	10199 (9558)	705 (851)
**Poverty profile**			
Low poverty incidence districts SES (m^2^)	877 (1499)	3533 (5279)	195 (442)
High poverty incidence districts (m^2^)	427 (323)	2055 (1694)	12 (20)

### Healthiness of supermarkets

As per Table 3, overall, the ratio (±SD) of the shelf area for unhealthy-to-healthy foods is 4 (2). From the table, all retail outlets assessed in the six districts were found to be less healthy. The ratio of ultra-processed to unprocessed or minimally processed foods was also 4.8 with a standard deviation of 1.4.

**Table 3:** Ratio of healthy/unprocessed/minimally processed foods to unhealthy/ultra-processed foods by district.

**District**	**Poverty profile**	**Unhealthy foods**	**Healthy foods**	**Ratio of unhealthy food: healthy food**	**Ultra-processed foods**	**Unprocessed/** **minimally processed foods**	**Ratio of ultra-processed foods: Unprocessed foods**
Accra Metro	Low	41,892	12,715	3.29	793	315	2.52
Ashiaman	Low	9,261	1,503	6.16	232	55	4.22
Ga South	High	15,003	2,241	6.70	354	61	5.80
La Nkwantanang	Low	100,259	18,627	5.38	625	150	4.17
Kpone katamanso	Low	18,168	7,071	2.57	252	38	6.63
Ningo Prampram	High	24,048	5,839	4.12	443	86	5.15
Overall (±SD),		34,772 (33,975)	7,999 (6,574)	4.70 (1.64)	450 (221)	118 (104)	4.75 (1.44)

## Discussion

Most of the modern retail outlets assessed were mini-marts. The most available food groups in the retail outlets were refined grains /grain products followed by sugar-sweetened beverages. Unprocessed staples, fresh meat, fish and poultry products, fresh vegetables and unsweetened beverages each occupied < 1% of the total shelf area allotted to food. Eighty-five percentage of the area occupied by food products in the modern retail outlets was allocated to unhealthy food categories. Almost $\frac{3}{4}$ of the food products were categorized as ultra-processed foods. Per ration of unhealthy-to-healthy foods or ultra-processed-to-unprocessed/minimally processed foods, all the retail outlets assessed were found to be unhealthy.

It has been previously reported that unhealthy foods are widely available in Ghana (9, 16, 28). As all outlets were assessed to be unhealthy overall, these outlets contribute to making ultra-processed and other unhealthy energy-dense, nutrient-poor foods more available. Notably, this study is cross-sectional and thus the findings of this study reflect the situation at a given time. However, all the retail outlets were assessed in a short period of time, so the findings are comparable within the sampled retail outlets.

Comparison of retail outlets and socio-economic status of districts using poverty headcount levels from the Ghana Statistical Service (29) shows important differences between the districts assessed. Although all the districts assessed in this study are located in the Greater Accra Region of Ghana, the most urbanized region of Ghana, the degrees of poverty incidence are clearly different. The number of supermarkets and mini-marts in Ga South (*n* = 11), a district with high poverty headcount, was modest compared to that in LANMA (*n* = 32), a district with low poverty incidence. A study conducted in Brazil (30) presented similar findings where lower socially deprived neighborhoods presented greater density of supermarkets. This may be because higher poverty incidence districts are less likely to attract high-end commercial establishments with a variety of food options because of their unstable urban infrastructure and lower consumer purchasing power.

In all the retail outlets assessed in Ga South, fresh fruits were not sold. Likewise, in Ashaiman, fresh vegetables and unsalted canned vegetables were not sold. This is similar to an earlier study in Ghana, where only five out of 13 supermarkets sold any fresh fruit or vegetables (23). Logistics such as transportation, preservation and storage of fresh food may be a barrier to the availability of fresh fruit and vegetables in the modern retail outlets assessed. Another explanation could be the status of supermarkets/mini-marts in the wider food environment. For instance, it is likely the role of these food retail outlets in supplying customers in Ghana with fresh fruit and vegetables is quite low due to the significance and usage of other retailers such as greengrocers or open markets (23). Also, the relatively small sizes of the supermarkets found in Greater Accra compared to supermarket sizes found in other studies, for example in the USA (20, 31) could account for the unavailability of fruits and vegetables in the supermarkets. This is expected as mini-marts and smaller supermarkets would assign less shelf space to all products given their limited space.

While supermarkets/mini-marts provide access to healthier foods such as whole grain cereals, legumes, oilseeds and nuts, fresh milk, fresh yogurt, sugar-free milk and fresh fruit juice, a larger shelf area was devoted to unhealthy foods. For instance, the NOVA classification showed that 68% of the food products identified in all the supermarkets assessed were ultra-processed. In addition, a study conducted in Brazil, a middle-income country, showed that about 50% of ultra-processed foods were purchased from super/hypermarkets and mini-marts (32). This confirms the conclusion drawn from previous studies in Ghana that energy-dense, unhealthy foods are widely available (9, 16, 22, 28). And that, widespread availability of unhealthy food is therefore an issue of concern since a relatively high availability of such foods can influence preference and consumption of energy-dense, nutrient-poor foods. Relative availability of products is a major determinant of purchasing decisions and thus consumption (33).

### Implications of findings

Our findings have implications for both researchers and policymakers. The widespread availability of ultra-processed foods (a proxy indicator of unhealthy foods) in the supermarkets/minimarts should be concerning to public health professionals. Governments, as well as the food industry, need to take actions. Policy makers seeking to improve health through dietary change, need to institute measures that improve relative availability of healthy, minimally processed foods within retail outlets. Such measures may include a mix of low-agency (fiscal policies, marketing restrictions) and high-agency (appropriate labeling) interventions.

### Limitations

While this study has some strengths as discussed, there are a number of important limitations to be noted. First, we recorded a high non-response rate. Forty-one percentage of the managers of the modern retail outlets we approached declined to participate in the study—citing COVID-19 pandemic-related reasons. Relatedly, a few other retail outlets were not operating and could not be accessed. This limited the number of retail outlets assessed which could have led to a possible selection bias. It is possible that the retail outlet that denied us access had healthier food categories than unhealthy ones or vice versa. The current study's operational definition of supermarkets and mini-marts may not be comparable with that used in high income countries, and thus limits comparability. Indeed, given that the study was delimited to food retail outlets in the Greater Accra Region, we will discourage any attempt to do such extrapolations or generalizations. In addition, the retail outlets assessed used different display formats. For instance, some displayed vegetables on four smaller shelves placed on top of each other (where we measured the shelf length and breadth of all four shelves) and others placed the vegetables in one high bin (where we only measured 1 shelf length and breadth); the former might appear to have more vegetables than the latter, while actually the latter might have more if the quantity was measured. However, this was a rare occurrence. Finally, this analysis did not take into consideration food product price. Food product price has been highlighted as one of the strongest drivers of food acquisition and consumption, were not taken. Finally, while available evidence shows a very strong correlation between ultra-processed foods and high content of free/added sugars, sodium and saturated fatty acids, we note that calorie density and level of processing are not foolproof indicators of healthiness or lack thereof.

## Conclusions

This study reveals widespread availability of unhealthy/ultra-processed foods in supermarkets/mini-marts in Greater Accra. For every 1 m^2^ of shelf area for healthy foods, there was 5 m^2^ of shelf area for unhealthy foods on average. For each unprocessed or minimally processed food product, there were 5 ultra-processed food products available in the supermarket/mini-mart on average. Relevant actors (the Food and Drugs Authority, Ministry of Food and Agriculture, Local Government, private sector, etc.) need to institute measures that improve relative availability of healthy, minimally processed foods within supermarkets/mini-marts.

## Data availability statement

The raw data supporting the conclusions of this article will be made available by the authors, without undue reservation.

## Ethics statement

The studies involving human participants were reviewed and approved by the Ghana Health Service Ethics Review Committee (GHS-ERC 005/06/19) and the University of Ghana Ethics Review Committee for the Humanities (ECH 152/18-19).

## Author contributions

AA and AL conceptualized the research question. AA performed the analysis and drafted the manuscript. GSA coordinated the data collection. WQ, AT, SV, and PA assisted in data collection. GSA, WQ, AT, RA, MH, CA, FZ, ML, KM, PA, DL, GA, DS, SV, and AL provided critical feedback, helped shape the research, analysis, and manuscript. AL supervised the work. All authors contributed to the manuscript and approved the submitted version.

## Funding

This study was supported by funding from the International Development Research Centre (IDRC) Food, Environment, and Health Program—IDRC, Canada—Grant Number: 108983-001. The funder, however, played no role in the study design, data collection, analysis or interpretation, or in writing the manuscript.

## Conflict of interest

The authors declare that the research was conducted in the absence of any commercial or financial relationships that could be construed as a potential conflict of interest.

## Publisher's note

All claims expressed in this article are solely those of the authors and do not necessarily represent those of their affiliated organizations, or those of the publisher, the editors and the reviewers. Any product that may be evaluated in this article, or claim that may be made by its manufacturer, is not guaranteed or endorsed by the publisher.

## Appendix

**Appendix 1:** The availability [measured by shelf area (m^2^)] of the different food items in the retail outlets assessed in the six.

**Districts**	**Accra Metro**			**Ashiaman**			**Ga South**			**La Nkwantanang**			**Kpone katamanso**			**Ningo Prampram**			
**Statistics**	**Mean**	**Std. Dev**	**Sum**	**Mean**	**Std. Dev**	**Sum**	**Mean**	**Std. Dev**	**Sum**	**Mean**	**Std. Dev**	**Sum**	**Mean**	**Std. Dev**	**Sum**	**Mean**	**Std. Dev**	**Sum**	**Total sum**
Refined.grains
tblbreakrefined.grain. products	1,129	1,172	15,799	702.5	291.5	4,215	435.0	229.0	3,915	1679.1	2310	36,941	1,139	2,208	6833.7	1,116	1043.2	11161.4	78865.1
Sugar. beverages	761	623	10,650	549.0	181.1	3,294	392.4	207.2	3,532	1122.9	1662	24,703	610	1,046	3662.2	692	520.6	6923.5	52764.3
Alcoholic beverages	239	284	3,345	136.2	181.8	817	760.5	1,644.8	6,845	658.8	1066	14,494	52	69	312.4	198	273.9	1975.5	27788.5
Sweet foods	287	199	4,023	82.8	76.9	497	49.1	46.5	442	303.4	562	6,676	362	839	2173.9	156	97.8	1563.0	15374.4
Milk. products	196	146	2,744	85.4	79.9	513	65.7	60.2	591	267.9	644	5,894	411	936	2466.3	301	327.6	3014.8	15222.7
Water	159	193	2,227	103.4	103.0	620	131.5	82.3	1,184	264.8	530	5,826	110	244	657.9	148	120.6	1483.3	11997.6
Fats/Oil product	140	138	1,955	20.8	26.7	125	17.2	26.8	155	288.0	548	6,337	246	588	1474.7	97	68.9	972.4	11018.4
Processed Meat, fish and poultry	146	129	2,042	34.7	24.9	208	11.0	22.6	99	210.2	373	4,625	307	691	1843.2	88	55.1	875.6	9693.7
Salted snack	179	280	2,505	9.0	10.6	54	1.8	4.1	16	175.4	227	3,859	260	579	1561.9	32	24.8	322.6	8318.3
Legumes, oilseeds and nuts	175	229	2,448	34.5	66.5	207	13.3	24.6	120	59.3	84	1,304	239	563	1436.7	17	14.0	174.6	5690.4
Whole grain cereals	75	77	1,051	16.2	17.9	97	11.8	13.3	106	154.2	270	3,392	45	58	268.3	51	68.8	509.8	5423.3
Fresh.fruit.juice	98	85	1,373	9.7	15.1	58	0.0	0.0	0	69.4	150	1,527	343	738	2057.3	24	30.7	240.3	5255.9
Canned vegetables with added salt	75	84	1,056	8.5	20.7	51	0.0	0.0	0	119.3	330	2,624	51	101	306.1	25	53.0	254.4	4291.3
Other	278	377	3,897	0.0	0.0	0	0.0	0.0	0	0.0	0	0	0	0	0.0	0	0.0	0.0	3897.2
Unsweetened.beverages	164	168	2,301	0.0	0.0	0	0.0	0.0	0	0.0	0	0	0	0	0.0	0	0.0	0.0	2301.1
Fresh vegetables and unsalted canned vegetables	64	111	902	0.0	0.0	0	1.0	2.9	9	25.6	91	563	121	297	728.5	1	2.3	7.1	2208.9
Fresh.fish. poultry	35	66	485	1.3	3.2	8	12.2	16.6	109	29.3	77	645	31	71	185.0	42	78.6	416.7	1848.7
Processed staples	37	45	517	0.0	0.0	0	0.0	0.0	0	0.0	0	0	0	0	0.0	0	0.0	0.0	516.9
Unprocessed staples	6	18	86	0.0	0.0	0	0.0	0.0	0	1.8	9	40	0	0	0.0	0	0.0	0.0	126.0
